# Easy and Rapid Purification of Highly Active Nisin

**DOI:** 10.1155/2011/175145

**Published:** 2011-09-18

**Authors:** André Abts, Antonino Mavaro, Jan Stindt, Patrick J. Bakkes, Sabine Metzger, Arnold J. M. Driessen, Sander H. J. Smits, Lutz Schmitt

**Affiliations:** ^1^Institute of Biochemistry, Heinrich Heine University Düsseldorf, Universitätsstrabe 1, 40225 Düsseldorf, Germany; ^2^Biological and Medical Research Center, Heinrich Heine University Düsseldorf, Universitätsstrabe 1, 40225 Düsseldorf, Germany; ^3^Department of Molecular Microbiology, Groningen Biomolecular Sciences and Biotechnology Institute, Zernike Institute for Advanced Materials and the Kluyver Centre for the Genomics of Industrial Microorganisms, University of Groningen, Nijenborgh 7, 9747 AG Groningen, The Netherlands

## Abstract

Nisin is an antimicrobial peptide produced and secreted by several *L. lactis* strains and is specifically active against Gram-positive bacteria. In previous studies, nisin was purified via cation exchange chromatography at low pH employing a single-step elution using 1 M NaCl. Here, we describe an optimized purification protocol using a five-step NaCl elution to remove contaminants. The obtained nisin is devoid of impurities and shows high bactericidal activity against the nisin-sensitive *L. lactis* strain NZ9000. Purified nisin exhibits an IC_50_ of ~3 nM, which is a tenfold improvement as compared to nisin obtained via the one-step elution procedure.

## 1. Introduction

The capacity to produce antimicrobial peptides (AMPs) is widespread among Gram-positive bacteria. These AMPs are directed against competitive microorganisms in order to generate a selective advantage for the producer organism [1]. AMPs can be divided in three major classes. Class I consists of the so-called lantibiotics, which are posttranslationally modified peptides containing (methyl-)lanthionines, and have a typical size of <5 kDa. Class II comprises heat-stable, nonmodified peptides of 37–58 amino acids (typical size of <10 kDa) with the leader peptide, as for Class I AMPs, being removed during maturation. Class III contains AMPs that are heat labile and that are generally larger in size (roughly 30 kDa). Especially heat-stable peptides secreted by lactic acid bacteria have been studied extensively because of their potential use as natural preservatives in the food industry. 

One of the best characterized AMPs is nisin, which is secreted by *Lactococcus lactis *and is a member of the Class I AMPs (for comprehensive reviews, see [2, 3]). Although nisin has been used as a food preservative for more than fifty years, no significant bacterial resistance against nisin has been reported [4]. Nisin, encoded by the *nisA* gene, is a (methyl-)lanthionine ring containing peptide that is ribosomally synthesized as a prepeptide consisting of 57 amino acids. The NisA prepeptide is modified posttranslationally by the dehydratase NisB, which selectively dehydrates Serine and Threonine residues, and by the cyclase NisC, which catalyzes lanthionine ring formation. NisT finally transports the modified prenisin across the cell membrane, and mature, biologically active nisin is produced upon cleavage of the leader peptide by the extracellular, membrane-anchored protease NisP. Mature nisin harbors three dehydrated amino acids (one dehydrobutyrine and two dehydroalanines), one lanthionine ring, and four methyl-lanthionine rings. In particular, these intramolecular rings are important for the biological activity of nisin [5]. It is worth mentioning that nisin induces its own synthesis via interaction with the two-component regulatory system NisRK.

Nisin is active against Gram-positive bacteria and exerts two killing mechanisms. Firstly, nisin inhibits cell-wall synthesis by binding to lipid II, an essential membrane-anchored cell-wall precursor, and secondly, nisin permeabilizes the target membrane. The binding of nisin to lipid II induces membrane integration of nisin resulting in the formation of a pore, likely composed of eight nisin and four lipid II molecules [6]. This highly specific interaction with lipid II is reflected by the low nano- to micromolar concentrations of nisin, sufficient to permeabilize the membrane of the target cells [7].

The bactericidal activity of AMPs is generally measured by quantifying growth inhibition of an AMP-sensitive target organism, grown either on agar plates or in liquid culture. On agar plates, zones of growth inhibition of the indicator organism can be easily visualized, and these so-called halo assays allow determination of the minimum inhibitory concentration (MIC) of the tested AMP [8]. Alternatively, bacterial growth can be monitored in liquid culture in dependence of the AMP concentration, by measuring the optical density. This method allows the simple determination of both the MIC and the IC_50_, that is, the concentration of AMP that inhibits cell growth by 50% [9].

Nisin from *L. lactis*, like almost all naturally produced AMPs, can be purified directly from the culture medium [10, 11]. It is a cationic peptide, and therefore, commonly purified using cation exchange chromatography (cIEX) at acidic pH, using high salt concentration for elution, typically a single-step elution with 1 M NaCl [12–16]. By using nisin purified via such a method, the IC_50_ and MIC values were determined for a variety of bacteria, such as various *L. lactis* strains, *Enterococcus faecium*, *Bacillus anthracis*, *Bacillus subtilis*, *Staphylococcus aureus, *and* Streptococcus mutans* [9, 17]. Whereas *E. faecium* and *S. mutans* were equally sensitive to nisin exhibiting an IC_50_ of 5 *μ*M and MIC of 12.5 *μ*M, *L. lactis* HP was much more sensitive to nisin, with an IC_50_ of 14 nM and MIC of 32 nM [9]. Nisin shows promising activity towards clinical isolates of the Methicillin-resistant* Staphylococcus aureus* (MRSA) bacterium, *Streptococcus pyogenenes,* and several of the most severe human pathogens, including the multiresistant *Streptococcus pneumoniae* and vancomycin-resistant *E. faecium* or *E. faecalis*, against which new effective antibiotics are most urgently needed [18–20]. In all these, studies purified nisin was used and the bactericidal activity of nisin was measured by determining the MIC or IC_50_.

Here, we describe a rapid and easy nisin purification protocol, optimized to yield active, monomeric nisin. For this purpose, nisin was either produced from *L. lactis* strain NZ9700 or purchased in the form of a lyophilized powder that contains 2.5% (w/w) nisin. Nisin from both sources was purified and tested for bactericidal activity using the nisin-sensitive *L. lactis* NZ9000 strain. During the purification low molecular weight contaminants are removed, which results in purified nisin with high specific activity. This allows a more accurate and reproducible determination of the biological activity of nisin. 

## 2. Material and Methods

### 2.1. Production and Purification of Nisin

#### 2.1.1. Nisin Produced by *L. lactis*

A culture of the *L. lactis* strain NZ9700 was grown overnight in M17 medium containing 0.5% (w/v) glucose (GM17) at 30°C. Next, 100 mL of the overnight culture was used to inoculate 2 l of GM17, and cells were grown at 30°C. At an OD_600_ of 0.8, the culture was supplemented with nisin (Sigma) to a final concentration of 1 ng/ml cell culture to ensure nisin production and growth was continued over night. Next, cells were removed by centrifugation (30 min., 6000xg at 4°C). The supernatant was used and diluted with 1 l of 50 mM lactic acid pH 3 resulting in a 3 l solution with a pH of 5. This solution was loaded on a 5 mL HiTrap SP HP cation exchange (cIEX) column (GE Healthcare) using a flow rate of 4 mL/min. Due to the large volume, this step is optimally performed overnight. Protein elution was monitored by measuring the absorbance at 215 nm. Unfortunately, it is not possible to detect nisin at 280 nm, because it does not contain any aromatic amino acids; therefore, the 215 nm was chosen as wavelength. The column was subsequently washed with 50 mM lactic acid pH 3 until a stable baseline was reached to remove nonspecifically bound material. Peptides were eluted by increasing the NaCl concentration stepwise using a flow rate of 1 mL/min, resulting in elution fractions with 200 mM (Step I), 400 mM (Step II), 600 mM (Step III), 800 mM (Step IV) and 1 M (Step V) NaCl. To remove NaCl, protein in the elution fractions was precipitated with 20% (v/v) trichloroacetic acid (TCA) overnight at 4°C. Precipitated protein was washed two times with ice-cold acetone to remove residual TCA. Finally, the protein pellet was suspended in 50 mM lactic acid pH 3. Nisin concentrations were determined by a colorimetric assay (Pierce BCA Protein Assay Kit, Thermo scientific) by measuring the absorbance at 584 nm according to the protocol of the manufacturer.

#### 2.1.2. Purchased Nisin

Commercial nisin (Sigma) is available as a lyophilized powder containing *∼*2.5% (w/w) nisin. 1.3 g of nisin powder (corresponding to 32.5 mg nisin) was dissolved in 100 mL 50 mM lactic acid pH 3 and filtered through a 0.45 *μ*m membrane filter (Pall Corporation). The nisin solution was applied to a 5 mL HiTrap SP HP cation exchange column (GE Healthcare) at a flow rate of 2 mL/min, whereas elution was performed at a flow rate of 1 mL/min. Nisin purification, precipitation, and concentration determination were carried out as described above.

### 2.2. Tricine-SDS-PAGE

Tricine-SDS-PAGE was essentially carried out as described in [21]. For analysis, 16 *μ*L sample was supplemented with 4 *μ*L 5x SDS sample buffer (0.2 M Tris-HCl, pH 6.8, 10% (w/v) SDS, 40% (v/v) glycerol, 0.02% (w/v) bromophenol blue, and 10 mM DTT) and loaded on a tricine gel consisting of a stacking gel containing 5% acrylamide and a separation gel containing 16% acrylamide. The gel was run at 100 V for 2 hours, and proteins were detected via silver staining. For all purification fractions, 3.2 *μ*g of total protein was analyzed.

### 2.3. MALDI-TOF Mass Spectrometry

Samples obtained from cIEX chromatography were precipitated with TCA and washed with acetone as described above. The protein pellets were then dried for 15 min. at 30°C in a vacuum concentrator (Eppendorf concentrator plus). Dried pellets were stored at −20°C until analysis. For mass spectrometric analysis, the samples were dissolved in water with 1% (v/v) formic acid. Measurements were performed using a MALDI-TOF instrument (Voyager-DE STR, Applied Biosystems) with a nitrogen laser (*λ* = 337 nm) operating in reflector mode with 25 kV acceleration voltage. The samples were prepared by the standard dried-droplet procedure, by applying 0.5 *μ*L of 2,5-dihydroxybenzoic acid (DHB) matrix solution (10 mg in 1 mL water) plus 0.5 *μ*L of sample solution. The droplet was dried by a gentle flow of air. An external calibration with the monomer ion of des-Pro-Bradykinin, Sub P, Bombesin, and Melittin was used. The spectrum was obtained by averaging 200 laser shots.

### 2.4. IC_50_ Determination of Nisin

To investigate the antimicrobial activity of nisin, the nisin sensitive *L. lactis* strain NZ9000 [22] was grown in a 96-well plate in GM17 medium. The total volume in each well was 200 *μ*L, consisting of 50 *μ*L sample and 150 *μ*L GM17 containing *L. lactis* NZ9000 cells (starting OD_600_ = 0.1). Samples were prepared by diluting nisin obtained from various cIEX elution fractions in 50 mM lactic acid pH 3 to yield final protein concentrations ranging from 0.15 nM to 300 nM in the wells. 50 mM lactic acid pH 3 without protein served as positive growth control. Cells were grown at 30°C, and the optical density was monitored at 620 nm every 20 min for a period of 8 hours (96 plate reader BMG). To determine the IC_50_ values, the optical density was normalized and plotted against the log of the nisin concentration. Data were evaluated according to

(1) $$Y=\frac{\text{OD}\;\min\;+\left(\text{OD}\;\max\;-\text{OD}\;\min\;\right)}{1+10^{\left((\log\;\;\left(\text{IC}50\right)-X)*\text{slope}\right)}}.$$

The OD_max_ value describes the normalized OD_600_ value in the starting plateau. The OD_min_ value corresponds to the normalized OD_600_ of the end plateau value. *Y* stands for the normalized optical density value, and *X* represents the logarithmic concentration of the peptide. The IC_50_ value is calculated as the value of the peptide concentration used where the growth inhibition (or OD_600_) is 50%. This corresponds to the inflection point of the resulting curves.

### 2.5. Growth Inhibition Visualized by a Halo Assay

The antimicrobial activity of the different nisin preparations was assessed by means of a halo assay. Purified nisin obtained from the different cIEX elution fractions were supplemented (16 *μ*L) with 4 *μ*L of 5x SDS sample buffer and tricine-SDS-PAGE was carried out as described above. After electrophoresis the gel was incubated for 30 min in an aqueous solution containing 20% (v/v) isopropanol and 10% (v/v) acetic acid. Subsequently, the gel was washed two times for 30 min in ddH_2_O. At this stage, the gel was kept in ddH_2_O at 8°C until usage. Finally, the gel was overlaid with GM17-agar (0.5% w/v agar) containing *L. lactis* NZ9000 cells at an OD_600_ of 0.1. After solidification, the overlaid gel was incubated overnight at 30°C to allow for bacterial growth. The bactericidal activity of nisin is readily visualized by the presence of clear zones (halos) resulting from growth inhibition.

## 3. Results

### 3.1. Purification of Nisin

#### 3.1.1. Lyophilized Commercial Nisin

To determine the antimicrobial activity of commercially available nisin, we used a lyophilized powder, which contains *∼*2.5% (w/w) nisin. To further purify nisin, we initially performed SP Sepharose cation exchange chromatography using 1 M NaCl to elute the bound nisin. Subsequent SDS-PAGE analysis of the eluate revealed a major protein band corresponding to a peptide with a molecular mass of about 3.5 kDa (data not shown) in line with the calculated mass of 3354 Da for mature nisin. However, several higher molecular weight components were also present. It is of note that these contaminants are not readily visualized by Coomassie Brilliant Blue staining, whereas these impurities are clearly detected by silver staining. Nisin purified via this method showed antimicrobial activity against *L. lactis* NZ9000 exhibiting an IC_50_ of 30 ± 12 nM.

**Figure 1:**
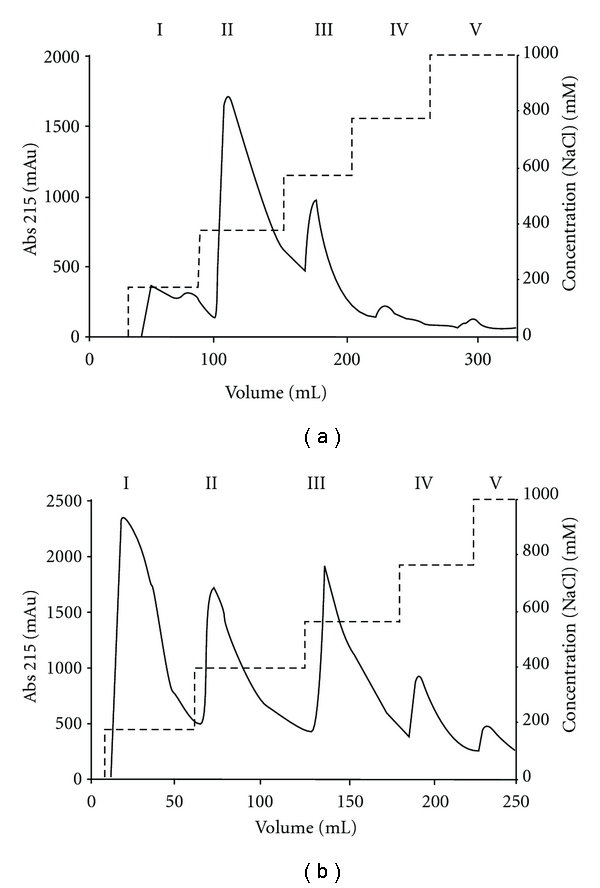
Purification of nisin via cation exchange chromatography. The elution profiles of the purification of commercial nisin (a) and nisin produced by *L. lactis* NZ9700 (b) are shown. In both cases, nisin is eluted from the column using a five-step gradient with 200 mM (Step I), 400 mM (Step II), 600 mM (Step III), 800 mM (Step IV) and 1 M NaCl (Step V). The different elution steps and corresponding NaCl concentrations are indicated by the dashed line and the right *y*-axis, respectively. Protein was detected by measuring the absorbance at 215 nm.

**Figure 2:**
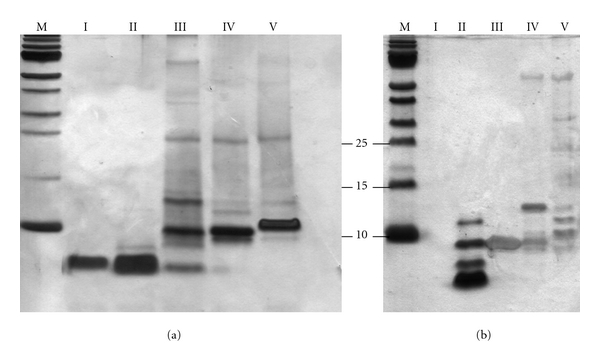
Tricine-SDS-PAGE analysis of the cIEX purification of nisin. Purification of commercial nisin (a) and nisin secreted by the *L. lactis* NZ9700 strain (b). M, marker proteins; I, elution with 200 mM NaCl; II, elution with 400 mM NaCl; III, elution with 600 mM NaCl; IV, elution with 800 mM NaCl; V, elution with 1 M NaCl. Protein was visualized by silver staining. The three lowest marker proteins are indicated with molecular weights (kDa).

The presence of contaminants prompted us to optimize the purification of nisin. We first tested elution with a linear gradient (50 times the column volume) ranging from 0–1 M NaCl. This approach, however, resulted in a broad peak eluting throughout the NaCl gradient and further analysis revealed no improvement when compared to the single-step 1 M NaCl elution (data not shown). In contrast, a substantial improvement was achieved when a five-step NaCl step gradient was used to elute nisin from the cIEX column (Figure 1(a)). Bound protein eluted at every step as evidenced by the elution profile and subsequent tricine-SDS-PAGE analysis (Figure 2(a)). The Step II elution fraction contained the bulk of nisin as evident by the major protein band with a corresponding molecular mass of *∼*3.5 kDa, whereas substantially lower amounts of nisin were detected in elution fractions I, III, and IV. In the Step V fraction no nisin was visible. The Step I and II elution fractions contained exclusively nisin, while elution fractions III–V contained predominantly higher molecular weight compounds (ranging from 6 kDa–70 kDa). In the latter fractions compounds with molecular weights of *∼*8 kDa and *∼*10 kDa were most prominent. The total protein concentration of the elution fractions was determined to be: 2.1 mg/mL for Step I (200 mM NaCl), 7.5 mg/mL for Step II (400 mM NaCl), 1.9 mg/mL for Step III (600 mM NaCl), 0.5 mg/mL for Step IV (800 mM NaCl) and 0.4 mg/mL for Step V (1 M NaCl) (Table 1). In Step II, *∼*60% of the total purified nisin eluted. Thus, it appears that 400 mM NaCl is sufficient to elute the vast majority of the nisin molecules. More importantly, the nisin eluting under these conditions is essentially devoid of contaminants.

#### 3.1.2. Nisin Produced by *L. lactis*

An alternative to purchasing nisin is to produce it in the laboratory, since *L. lactis* strains that secrete nisin in large amounts are readily available. We used *L. lactis* NZ9700 grown in GM17 medium to produce nisin (see Section 2). Culture supernatant containing nisin was subjected to cIEX chromatography using the same five-step NaCl elution gradient as described above. Here, a high absorbance at 215 nm occurred at the Step I elution (Figure 1(b)), but this did not correspond to nisin or other proteins as evidenced by silver-staining following SDS-PAGE analysis (Figure 2(b), lane 2). Likely, this high absorbance is due to ingredients from the growth medium, which contains large amounts of peptone, tryptone, and yeast extract. This was confirmed by a run with only GM17 media (data not shown). The remainder of the elution profile is similar to that of the lyophilized nisin purification, with two major absorbance peaks observed for the Step II and III fractions (Figure 1(b)). Analysis of the protein content of the different elution fractions revealed that nisin is exclusively found in the 400 mM NaCl elution fraction (Figure 2(b)). The total protein concentration of the 400 mM elution fraction was 2.9 mg/mL. However, whereas nisin is most prominent in this fraction, components with a MW *∼*6 kDa, *∼*10 kDa, and *∼*12 kDa are also present. The 0.6–1 M elution fractions on the other hand contained compounds with molecular weights ranging from 8 kDa–70 kDa, similar to those observed for the purification of lyophilized nisin (Figures 2(a) and 2(b)). When compared to the purification of lyophilized nisin, nisin purified from GM17 medium still contained contaminants. This difference in purity may relate to differences in the loaded material. The lyophilized nisin powder (also containing denatured milk solids) was dissolved in 50 mM lactic acid, whereas nisin produced by *L. lactis* NZ9700 was applied to the cationic exchange column as a 1 : 2 mixture of GM17 culture medium and 50 mM lactic acid, respectively.

We considered the possibility that the compounds with molecular weights of 6–8 kDa may represent the unprocessed form of nisin. Immature nisin, that is, nisin still containing the leader sequence, has a molecular weight of 5.9 kDa and may arise if inefficient leader cleavage by the protease NisP occurs. We therefore performed Western-blot analysis using a polyclonal antibody raised against the nisin leader sequence. Purified prenisin secreted by a *L. lactis* strain lacking NisP was used as a positive control for Western-blot analysis [23]. For all elution fractions, no signals were observed suggesting that the observed compounds are not derived from prenisin (data not shown).

### 3.2. Biological Activity of Nisin

**Figure 3:**
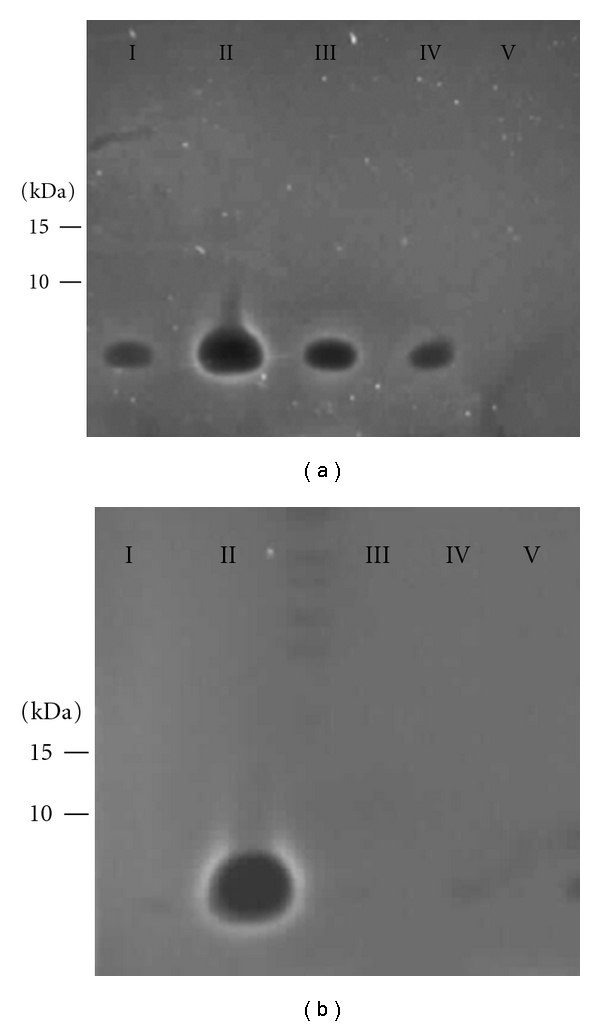
Bactericidal activity of the various nisin purification fractions. Equal amounts of protein of the different elution fractions (Step I–V) from the purification of commercial nisin (a) and from nisin secreted by the *L. lactis* NZ9700 strain (b) were run on a tricine-SDS-PA gel and overlaid with nisin-sensitive *L. lactis* NZ9000 cells (see Section 2). The position of marker proteins with known molecular weight (kDa) are indicated on the left. The growth inhibition zones are visible as dark areas. Lanes I–V represent the five different elution fractions of the cation exchange chromatography. For both purifications, maximum growth inhibition is observed for the Step II elution fraction (400 mM NaCl). Notably, the growth inhibition zone is only visible at a position of *∼*3.5 kDa.

To test the biological activity of the purified nisin, we used *L. lactis* NZ9000 as indicator organism. *L. lactis* NZ9000 is a derivative of the plasmid-cured *L. lactis* MG1363 and contains the *nisRK* genes inserted in the chromosomal *pepN* locus [24]. This strain is commonly used as the host for nisin-induced expression system (NICE) purposes [25]. However, since this strain lacks the nisin immunity genes *nisIFEG,* it is sensitive to nisin [22]. The antimicrobial activities associated with the different elution fractions obtained as described above were tested in a so-called halo assay. For this, the various fractions containing nisin were analyzed by tricine-SDS-PAGE and the tricine gel was overlaid with GM17-agar containing nisin sensitive bacteria (see Section 2). The biological activity of nisin is visualized by the growth inhibition zones (halos) at the position where nisin is present. The results for the lyophilized and the laboratory produced nisin are shown in Figures 3(a) and 3(b), respectively. After overnight incubation at 30°C halos were observed for elution Step I–IV for the lyophilized nisin, while for the produced nisin, a halo was only observed for elution fraction II. For both purifications, the highest level of growth inhibition was observed for fraction II. Importantly, the zones of inhibition are located only at the position of the 3.5 kDa nisin peptide (Figure 3). Thus, purified nisin was biologically active and no growth inhibitory activity is associated with the higher molecular weight compounds.

### 3.3. Mass Spectrometry

**Figure 4:**
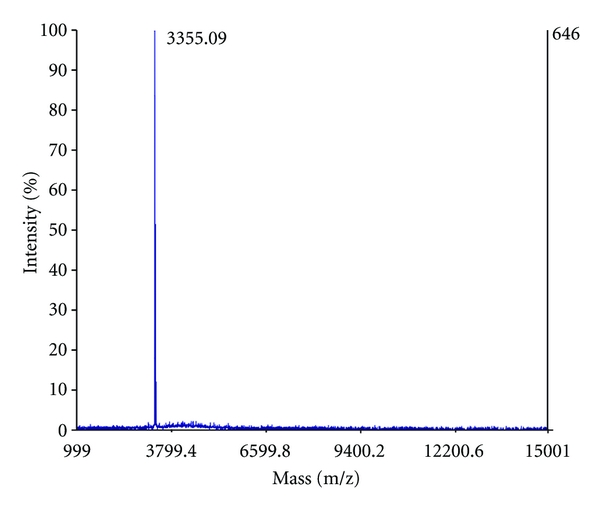
MALDI-TOF mass spectrometry analysis of purified nisin. Mass spectrum of the Step II elution fraction (400 mM NaCl) from the lyophilized nisin purification (for corresponding tricine-SDS-PAGE analysis, see Figure 2(a), lane II).

To assess and confirm the presence of nisin in the individual cIEX elution fractions (Step II–V) of the lyophilized nisin purification, we applied MALDI-TOF mass spectrometry. The 400 mM NaCl elution fraction contained only one peptide with a molecular mass of 3355.09 Da (Figure 4), which is in agreement with the calculated mass of 3354.07 Da for nisin. Peak integration of the total mass spectrum revealed that the 400 mM elution fraction contains >98% of nisin, indicating that this fraction is essentially devoid of contaminants. Nisin was also found in the 600 mM and 800 mM elution fractions, whereas nisin was not detected in the 1 M NaCl fraction. These results are in agreement with tricine-SDS-PAGE analysis (Figure 2(a)).

However, the 0.6–1 M NaCl elution fraction contained several peptides with higher molecular masses. Subsequent tandem MS analysis of these peptides yielded in-sequence tags of eight amino acids and six amino acids, which unfortunately could not be assigned to specific proteins. This was due to the fact that the obtained sequence tags were too short and when blasted gave multiple different protein hits (data not shown). Nevertheless, the obtained sequence tags did not match with the primary sequence of (pre)nisin. Therefore, it can be excluded that the peptide contaminants with molecular masses of *∼*6–8 kDa are derived from prenisin.

### 3.4. Antimicrobial Activity of Purified Nisin

**Figure 5:**
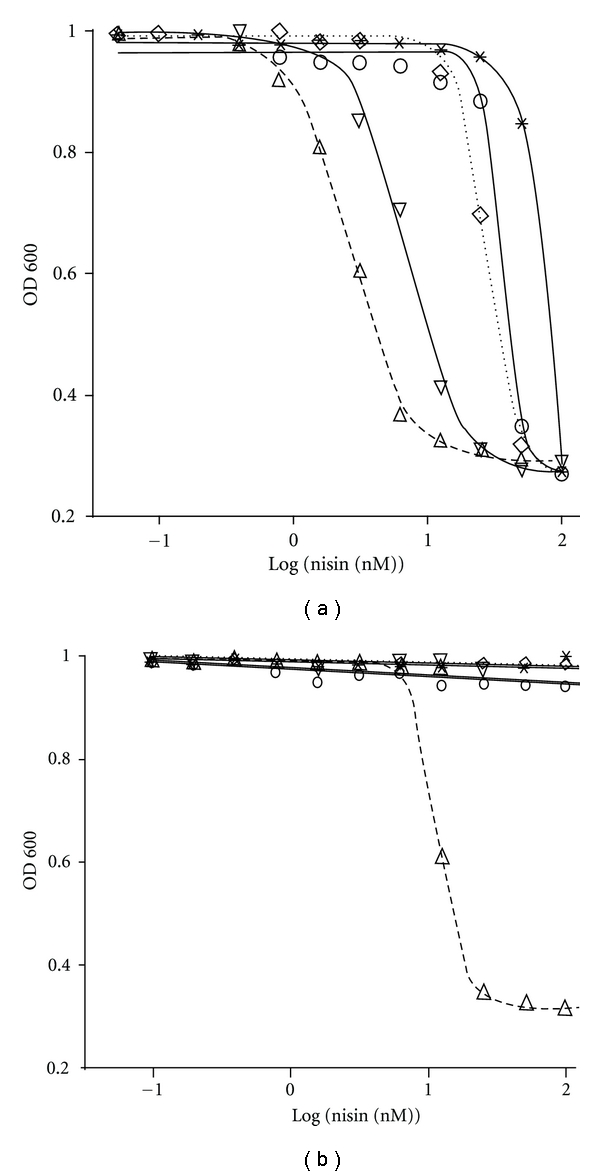
IC_50_ determination of the nisin purification fractions. Growth inhibition experiments were performed with nisin obtained from the different elution fractions of the purifications of commercial nisin (a) and of nisin secreted by the *L. lactis* NZ9700 strain (b). The log of the used nisin concentration of each elution fraction is plotted against the normalized optical density of *L. lactis* NZ9000 after five hours of growth. Shown are the inhibition curves for the NaCl elution fractions of 200 mM (o), 400 mM (∆), 600 mM (*∇*), 800 mM (*◊*), and 1 M (*). Data was fitted and evaluated according to (1).

To quantitatively assess the growth inhibitory activity of nisin obtained from the different purification fractions, a liquid culture assay was performed using *L. lactis* NZ9000 as reporter organism. The optical density of the *L. lactis* NZ9000 cultures after 5 hours of growth was plotted against the total protein concentration of the nisin purification fractions. Results are shown for both, the lyophilized (Figure 5(a)) and the produced nisin (Figure 5(b)), respectively. The resulting growth curves and the calculated IC_50_ values are shown in Figure 5 and Table 1, respectively. 

For lyophilized nisin, all cIEX elution fractions exhibited growth inhibitory activity, however, with very distinct IC_50_ values (Figure 5(a) and Table 1). The Step II elution fraction displayed the highest bactericidal activity with an IC_50_ of 2.6 ± 0.1 nM. The other elution fractions showed substantially higher IC_50_ values. Whereas the Step V elution fraction had only an inhibitory effect at the highest tested concentrations, the Step I, III, and IV elution fractions exhibited an IC_50_ value of 35.1 ± 0.1 nM, 6.9 ± 0.2 nM, and 27.0 ± 0.2 nM, respectively. Thus, the 400 mM NaCl elution fraction contains not only the bulk of nisin, it also contains nisin that displayed the highest specific activity. 

A similar observation can be made for nisin purified from the medium (Figure 5(b)). However, here only the 400 mM elution fraction shows bactericidal activity, which is in agreement with tricine-SDS-PAGE analysis (Figure 2(b)) and the halo assay (Figure 3(b)). The calculated IC_50_ of 11.2 ± 0.3 nM is, however, *∼*4-fold higher than that of the corresponding fraction obtained from the purification of lyophilized nisin. We attribute this difference to the contaminants that are still present (Figure 2(b), lane II). Nevertheless, for both purifications the highest bactericidal activity is associated with the fraction that contains the highest amount of nisin (Figures 2-3 and 5). Taken together the data indicate that nisin obtained from the Step II elution fraction has the highest specific activity. 

To determine whether NaCl used for elution has an effect on nisin activity, we repeated the experiment and adjusted the concentration of NaCl after elution in every fraction to 500 mM either by dilution with buffer without salt or by adding buffer and salt. Precipitated protein from these fractions was subsequently used for growth experiments as described above. In all cases, the IC_50_ values of the “salt experiment” were slightly higher than when measured directly after elution, indicating that more nisin is needed to inhibit cell growth by 50% (data not shown). This indicates that residual salt does not have a major influence on the activity of nisin. It can, therefore, be excluded that the differences in IC_50_ values of the various nisin containing elution fractions are induced by the amounts of NaCl used to elute nisin from the column.

## 4. Discussion

AMPs produced by Gram-positive bacteria form a unique group secreted peptides [1]. Their uniqueness of especially the lantibiotic group of AMPs, resides in the posttranslational modifications, such as dehydration of amino acids and intramolecular thioether bridges. One of the best-characterized AMP is nisin, a compound used for more than 40 years in up to 80 countries as an effective agent to combat food-borne pathogens. Nisin has been purified and its antimicrobial activity verified in numerous of studies [9]. Commonly, nisin and other cationic AMPs are purified using a single 1 M NaCl elution step, from a cIEX column at an acidic pH [12–14, 16, 26]. 

The activity of AMPs is strictly dependent on the target organism. For example, the IC_50_ value of nisin ranges from 14 nM for *L. lactis* HP to 5 *μ*M for Vancomycin-resistant *E. faecium, *with respective MICs of 32 nM and 12.5 *μ*M [9]. In all these studies, it is noticeable that there is a large variation in the sensitivity to nisin between isogenic strains of Gram-positive bacteria, whereas some bacteria are inherently resistant to nisin. There are several mechanisms by which bacteria can become resistant to an antibiotic. The most prominent example is the enzymatic destruction or modification of the antibiotic, thereby rendering it ineffective. *β*-Lactamases, for example, degrade the *β*-lactam ring of penicillins. A second important mechanism of resistance is shielding of the target such that the antibiotic cannot get access to it—for example, by cell-surface alterations (capsules S-layers) or by active extrusion by efflux pumps. Moreover, the resistance of the AMP producer organism towards its secreted AMP (autoimmunity) is typically based on ATP-binding cassette (ABC) transporters which expel the AMP from the membrane. 

In *L. lactis* NZ9700, cells autoimmunity is mediated by the scavenger protein NisI and the ABC transporter NisFEG [5]. *L. lactis* cells lacking the NisIFEG defense system (e.g., *L. lactis* NZ9000) are sensitive to nisin and can be used as indicator organism to measure the biological activity of nisin. The *nisI *and* nisFEG* genes are part of the *nis* operon and are expressed in concert with the genes involved in nisin production and secretion. Recently, in nisin-non-producing *L. lactis*, nisin resistance was shown to be conferred by a specific nisin resistance gene (*nsr*), which encodes a 35 kDa nisin resistance protein (NSR). NSR proteolytically removes the last six amino acids of nisin, thereby reducing its bactericidal activity by a factor of 100 [27]. 

The level of intrinsic resistance and the employed mechanisms of antibiotic resistance may differ greatly among microorganisms. It is, therefore, difficult, if not impossible, to directly compare IC_50_ values for a given AMP when comparing strains. Moreover, the purity of the AMP preparation is of great importance to determine accurately the bactericidal activity of the AMP. 

In this study, we optimized the purification of the AMP nisin and determined its IC_50_ values against the nisin-sensitive *L. lactis* strain NZ9000. Nisin typically purified via a 1 M NaCl one-step elution yields high levels of active nisin (see above and [28]) but contains a substantial amount of contaminants. We show that these contaminants, which are mainly proteinaceous in nature, are largely removed by using a five-step NaCl elution. Notably, 400 mM NaCl (Step II) was sufficient to elute the bulk of the nisin molecules, while the majority of contaminants remain bound to the column. In this manner, a nisin preparation was obtained that exhibited a high specific activity. When tested against the nisin-sensitive *L. lactis* NZ9000, this highly active nisin exhibited an IC_50_ of 2.6 ± 0.1 nM, which is a 10-fold improvement as compared to the nisin obtained via the one-step elution. The potent bactericidal activity of nisin against *L. lactis* NZ9000 lacking NisI and NisFEG suggests that these autoimmunity proteins are of great importance for *L. lactis* cells that produce nisin (e.g., *L. lactis* NZ9700).

AMPs get more and more into the focus of biochemical, biophysical, and medical studies due to their antimicrobial activity against a wide variety of bacteria. Here, we report an easy and rapid protocol for the purification of highly active nisin, purified either directly from the culture medium or from a commercially available lyophilized powder. Our studies demonstrate the importance of obtaining AMP preparations with high specific activity. A pure, homogenous, and biologically active preparation will ensure reliable determination of the efficacy of AMPs towards their microbial target(s). Due to the similar chemical and biophysical properties of lantibiotics, our manner of purification may also apply to AMPs other than nisin.

## Figures and Tables

**Table 1:** *IC*_50_ values of each nisin containing fraction eluted from the cIEX column. Values are combined data from at least three independent nisin purifications and subsequent inhibition experiments.

	Purchased nisin		NZ9700 secreted nisin	
Elution step	IC_50_ (nM)	Yield (mg)	IC_50_ (nM)	Yield (mg)
Step I (200 mM)	35.1 ± 0.1	4.2	n.i.	0.06
Step II (400 mM)	2.6 ± 0.1	15.1	11.2 ± 0.3	5.90
Step III (600 mM)	6.9 ± 0.2	3.8	n.i.	3.29
Step IV (800 mM)	27.0 ± 0.2	1.1	n.i.	0.14
Step V (1 M)	n.i.	0.8	n.i.	0.45

n.i = no inhibition observed under the experimental setup.

## Abbreviations

IC_50_: 50% Inhibitory concentration
MIC: Minimal inhibitory concentration
MW: Molecular weight
AMP: Antimicrobial peptide.
